# Genetic Engineering of VHH Antibody Fragments for Efficient Site-Specific Conjugation to Polysaccharides

**DOI:** 10.1021/acs.bioconjchem.5c00167

**Published:** 2025-05-23

**Authors:** Lin Zhong, Lisanne C. M. Morshuis, Michelle Koerselman, Angela Memelink, Anna Kolecka, Raimond Heukers, Theo Verrips, Marcel Karperien, Bram Zoetebier

**Affiliations:** † Department of Developmental BioEngineering, TechMed Institute, 3230 University of Twente, Enschede 7522 NB, The Netherlands; ‡ Orthros Medical BV, Drosteweg 8, 8101NB Raalte, The Netherlands; § QVQ Holding BV, Yalelaan 1, 3584CL Utrecht, The Netherlands

## Abstract

Site-selective modifications of proteins, without compromising their biological activity, are highly sought after due to their critical role in many biomedical applications. Here, we established a universal and efficient approach for site-selective conjugation of a variable domain of single-chain heavy-chain only antibody fragments (VHH) to polysaccharides using thiol–maleimide chemistry, known for its specificity and efficiency. This is achieved by genetically engineering an unpaired cysteine (Cys) residue in a C-terminal extension of VHHs. In this study, we synthesized two maleimide-functionalized polysaccharides, i.e., dextran-maleimide (Dex-Mal) and hyaluronic acid-maleimide (HA-Mal), for protein conjugation. Six distinct VHHs were selected and engineered with C-terminal extensions containing Cys residues for conjugation with Dex-Mal and HA-Mal. Conjugation efficiency varied among VHHs due to structural heterogeneity, which influenced the reactivity of the engineered Cys residues. One VHH, specific to TNFα (anti-TNFα-VHH), exhibited low conjugation efficiency (<20%); however, efficiency was fully restored when a flexible glycine-serine G_4_S linker was introduced between the variable domain and the C-terminal Cys tag. Additionally, incorporation of two free Cys residues in the C-terminal tail further enhanced conjugation efficiency. This work establishes a robust and versatile approach for generating protein–polysaccharide conjugates, paving the way for therapeutic and diagnostic applications.

## Graphical abstract

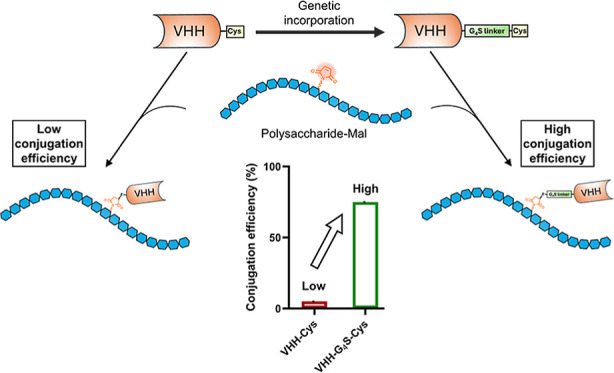

## 1 Introduction

Therapeutic proteins, including peptides, recombinant proteins, and monoclonal antibodies, have an important role in almost every field of medicine and have been proven highly successful in clinics for the treatment of a wide range of diseases including cancer, immunological, and infectious diseases and metabolic disorders. Since the first peptide therapeutic, recombinant human insulin, was approved by the FDA in 1982, over 300 approved protein drugs are currently on the market and more than thousands are in clinical development.^1^ The global protein therapeutics market has been continuously growing and is expected to reach the $400 billion market by 2026 at a compound annual growth rate (CAGR) of 8.3% for the forecast period of 2021 to 2026.^2^ Compared to small-molecule drugs, proteins often exhibit higher bioactivity and higher specificity due to the complex secondary and tertiary structures of proteins that cannot be easily mimicked by simple chemical compounds. Moreover, protein therapeutics are relatively safe due to their high specificity, which minimizes interference with normal biological processes and reduces adverse effects.^3^ Despite these advantages, the clinical application of protein therapeutics is still limited. Some of these proteins have short pharmacokinetic properties, often due to their molecular weight being below the threshold for a kidney filtration cutoff of around 45 kDa^4^ and the fact that they are subject to enzymatic degradation.^5^ Thus, the short circulation half-life of these therapeutics may require frequent administration of medication to maintain dosage within the therapeutic window, consequently increasing the risk of side effects and hampering patient compliance.

Current technologies with the goal of improving pharmacokinetic properties of proteins in vivo mainly aim at overcoming the body’s natural clearance by increasing the apparent molecular weight or the hydrodynamic radius of potential therapeutic proteins. Conjugation of highly soluble polymers to bioactive proteins is one of the many approaches for extending circulation half-life by reducing renal clearance.^6–8^ Among various commercially available polymers, PEG, a linear or branched polyether with hydroxyl end groups, has been the first and most used. Over the last three decades, more than 15 PEGylated protein drugs have been approved by the FDA and are highly successful in the clinic.^9^ However, due to the nonbiodegradability of PEG, polypeptides^10–12^ and polysaccharides^13,14^ have recently attracted more and more attention as alternatives. Additionally, polysaccharides, naturally occurring biopolymers with multiple reactive sites, enable the conjugation of bioactive proteins to single-chain biopolymers for multivalent protein conjugation. Previous studies have shown that such multivalent conjugates can enhance the potency and therapeutic function of tethered proteins compared to equimolar concentrations of their unconjugated counterparts; however, the amount of bioactive proteins that truly reach their target once they are conjugated might be reduced.^15,16^

Over the past few decades, various strategies have been described to attach therapeutic proteins to polymers to form protein–polymer conjugates. One common approach for producing polymer–protein conjugates relies on nonselective chemical reactions, predominantly targeting lysine residues that are highly prevalent on the surface of proteins.^17,18^ However, this results in poor site selectivity, which can potentially alter protein bioactivity or antigen-binding affinity if conjugation takes place on the active site.^19^ To overcome the low site selectivity of lysine conjugation, alternative methods have been developed, such as coupling to the thiol group of cysteine (Cys). However, in most native proteins, Cys residues are paired and involved in the formation of disulfide bonds determining secondary and tertiary protein structures and thus are not available for conjugation to a polymer. This issue can be solved by the introduction of an unpaired Cys into the protein of interest. Typically, an additional genetic engineering step is required to introduce a solvent-accessible unpaired Cys at the C- or N-terminus of the protein, which easily reacts with thiol-reactive groups such as maleimide.^20,21^ The reaction between the thiol group of Cys and maleimide derivatives proceeds rapidly under mild conditions with high selectivity. However, the structural heterogeneity of proteins, arising from differences in amino acid sequences, can influence the solvent accessibility of the unpaired Cys residues, leading to variations in the conjugation efficiency. Therefore, a universal strategy is required to achieve high conjugation yields and site-specific polymer attachment to the protein while maintaining its biological activity for potential biomedical applications.

Recently, the so-called variable domain of single-chain heavy-chain only antibody (VHH) fragments or nanobodies has gained popularity in biomedical applications as an alternative to the conventional monoclonal antibody due to their superior stability, easier and cost-effective production, and reduced immunogenicity.^22,23^ However, VHHs can be more easily removed by the kidneys and have a short half-life in the body of about 2 h, owing to the relatively low molecular weight of 12–15 kDa, which is a potential drawback of these molecules as therapeutic agents.^24,25^ For this reason, strategies based on the covalent conjugation of polymers to VHH were employed to increase the circulation half-life and duration of action of VHH. In contrast to conventional antibodies, the genetic engineering of VHHs is relatively straightforward.

Site-specific modification of VHHs via thiol–maleimide chemistry has become a well-established approach for improving the homogeneity, stability, and functionality of bioconjugates. Massa et al. demonstrated that introducing an unpaired cysteine residue at the C-terminus of VHHs enables efficient and selective conjugation to maleimide-functionalized chelators, producing radiotracers with preserved antigen-binding capacity and favorable in vivo imaging characteristics.^26^ Similarly, Aubrey et al. applied cysteine-based maleimide chemistry to engineer site-specific antibody–drug conjugates (ADCs) using trastuzumab-derived scFv fragments, achieving defined drug-to-antibody ratios and potent cytotoxic effects against HER2-positive breast cancer cells.^27^ While these studies underscore the advantages of site-directed bioconjugation for both imaging and therapeutic applications, they rely primarily on small-molecule payloads. In contrast, we extend this concept by enabling site-specific conjugation of VHHs to biodegradable polysaccharides, such as dextran and hyaluronic acid, offering a modular and multivalent platform to enhance the circulation half-life and therapeutic efficacy of nanobody-based constructs.

Taking all of these aspects into account, we synthesized two maleimide-functionalized polysaccharides: dextran maleimide (Dex-Mal) and hyaluronic acid maleimide (HA-Mal), for the generation of VHH-polymer (VHH-Dex or VHH-HA) conjugates. Six different VHHs, genetically engineered to include an unpaired Cys in the C-terminal tail, were selected and produced for conjugation to the polymers. The conjugation efficiency varied considerably in a sequence-dependent manner, with one VHH (anti-TNFα-VHH) showing an extremely poor conjugation efficiency. This limitation was addressed by genetically introducing a hydrophilic, flexible glycine-serine G_4_S linker between the functional domain of the VHH and the C-terminal tag with unpaired Cys.

Intriguingly, incorporating an additional unpaired Cys residue into the C-terminal tail further enhanced the conjugation efficiency. Furthermore, the binding affinity of VHHs to their target protein after modification was evaluated.

## 2 Materials and Methods

### 2.1 Materials

Dextran (40 kDa, pharmaceutical grade) was purchased from Pharmacosmos, Holbæk, Denmark. Sodium hyaluronate (27 kDa, pharmaceutical grade) was purchased from Contipro Pharma, Dolní Dobrouč, Czech Republic. *n*-Boc-1,4-diaminobutane (≥97.0%), *p*-nitrophenyl chloroformate (PNC, 96%), LiCl (≥99.0%), anhydrous dimethylformamide (DMF, 99.8%), pyridine (anhydrous, 99.8%), sodium hydroxide (NaOH, ≥97.0%), NaHCO_3_ (99.7%), NaCl (≥99.0%), DMSO-*d*_6_ (99.9%), trifluoroacetic acid (TFA, ≥99.0%), D_2_O (99.9 atom % D), tris­(2-carboxyethyl)­phosphine hydrochloride (TCEP), MES monohydrate (99.0%), Amicon Ultra-0.5 centrifugal filter (3 kDa MWCO), and horseradish peroxidase (HRP, 325 units/mg solid) were purchased from Sigma-Aldrich. 4-(4,6-Dimethoxy-1,3,5-triazin-2-yl)-4-methylmorpholinium chloride (DMTMM, 97.0%) and *n*-(α-maleimidoacetoxy) succinimide ester (AMAS, 95.0%) were purchased from Fluorochem Ltd., Hadfield, UK. *N*-(2-Aminoethyl)­maleimide hydrochloride (AEM·HCl, >93.0%) was purchased from TCI Europe NV Zwijndrecht, Belgium. Ethanol (≥99.9%) and diethyl ether (≥99.7%) were purchased from Merck, Kenilworth, NJ, USA. Phosphate buffered saline (PBS) was obtained from Lonza, Basel, Switzerland. Milli-Q water was used from the Milli-Q Advantage A10 system (Merck KGaA, Darmstadt, Germany) equipped with a 0.22 μm Millipak-40 Express filter. Caution! Trifluoroacetic acid is highly corrosive (GHS 1A) and causes severe burns; handle in a fume hood with full protective equipment. Sodium hydroxide is strongly caustic (GHS 1A) and causes serious skin and eye damage. Dimethylformamide is a reproductive toxin (GHS 1B) and harmful through skin absorption; use gloves and work in a fume hood.

### 2.2 Synthesis of HA-Mal

HA-Mal was prepared by amidation of the HA carboxyl groups with *N*-(2-aminoethyl)­maleimide by using a procedure adapted from Rydergren^28^ and Ding et al.^29^ Briefly, sodium hyaluronate (5.00 g, 12.5 mmol repeating units (r.u.)) was dissolved in 160 mL of MES solution (20 mM, pH 6.8) in a 250 mL round-bottom flask equipped with a stirrer bar. While stirring at room temperature, 4-(4,6-dimethoxy-1,3,5-triazin-2-yl)-4-methylmorpholinium chloride (DMTMM, 1.18 g, 4.26 mmol) and *N*-(2-aminoethyl)­maleimide hydrochloride (AEM·HCl, 0.139 g, 0.79 mmol) were added subsequently. The mixture was stirred at room temperature for 24 h. The addition of DMTMM and AEM·HCl was repeated 2 times, including 24 h reaction time. Afterward, 40 mL of saturated NaCl solution was added to the reaction mixture, and the reaction mixture was poured into 2.5 L of cold ethanol. The crude product was isolated by centrifugation at 5000 rpm followed by drying in vacuum. The crude product was dissolved in 75 mL of Milli-Q water and dialyzed against Milli-Q water for 3 days (MWCO 3500 Da). Lyophilization yielded the product as a white foam (4.61 g, 91% yield). The successful synthesis of HA-Mal was confirmed using ^1^H NMR in D_2_O. ^1^H NMR (400 MHz, D_2_O): δ­(ppm) = 1.98 (acetyl CH_3_, s, 3H); 3.2–4.2 (saccharide ring, m, 10H); 4.34 (s, 1H); 4.43 (d, 1H); 6.79 (maleimide protons, s, 2H). The number of maleimides per 100 disaccharide units was calculated based on the acetyl group at 1.98 ppm (corresponding to the 3 methyl protons from HA), compared with the integral of the 2 maleimide protons at 6.79 ppm. The resulting HA-Mal contained 12 maleimide moieties per 100 disaccharide units (Figure 1).

**Figure 1:**
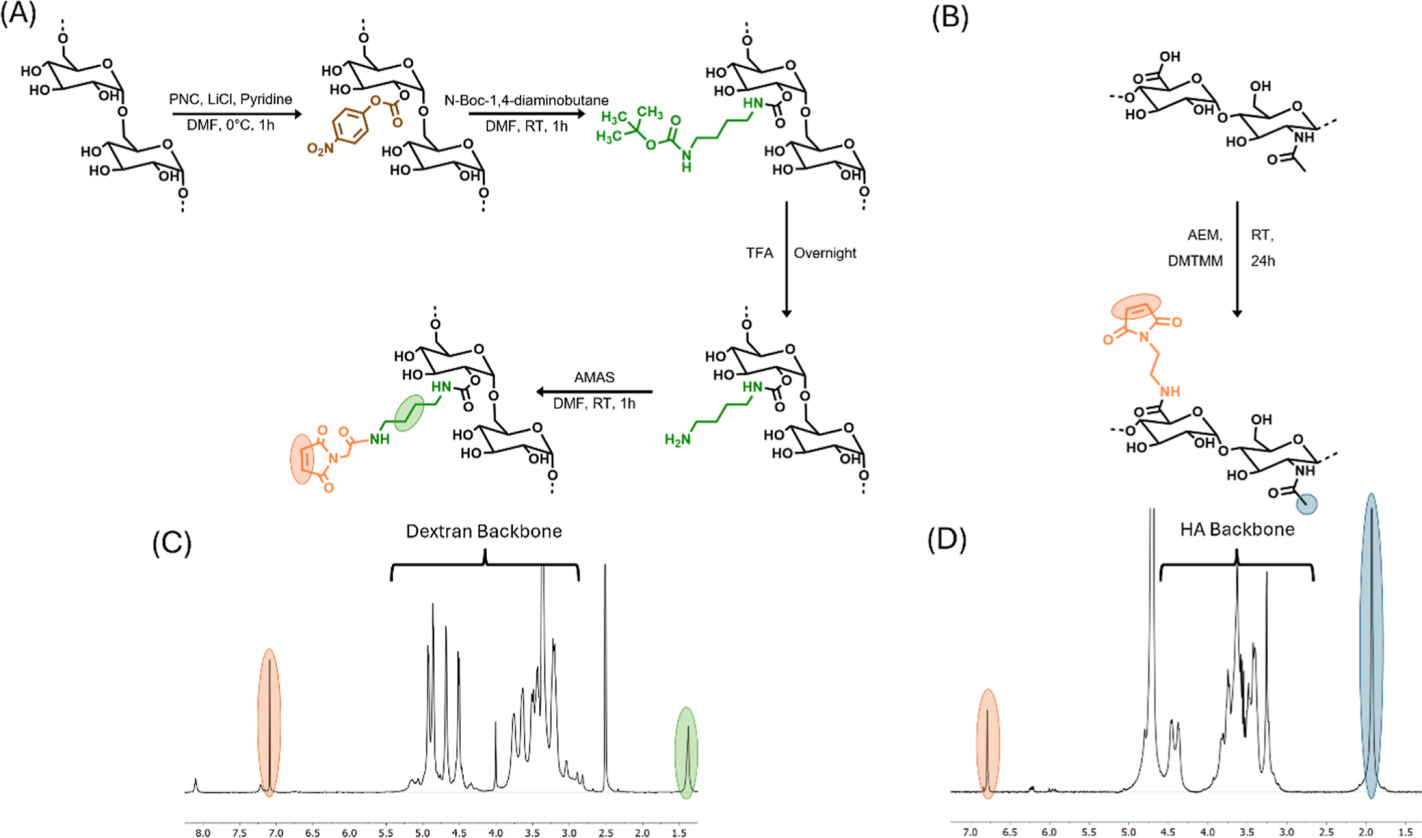
Synthesis route of Dex-Mal (A) and HA-Mal (B) and the ^1^H NMR spectra of the corresponding products Dex-Mal measured in DMSO-*d*_6_ (C) and HA-Mal measured in D_2_O (D). The backbone and characteristic peaks are indicated; maleimide protons in orange, butyl protons in green, and acetyl protons in blue.

### 2.3 Synthesis of Dex-Mal

Dex-(bNH): Dextran was activated with *para*-nitrophenyl chloroformate (PNC), as previously described.^30,31^ Typically, LiCl (4.0 g, dried at 115 °C) and dextran (5.0 g, 30.8 mmol r.u.) are weighed into a 500 mL three-neck round-bottom flask equipped with a stirrer bar. The flask is evacuated and refilled with nitrogen 3 times after which it is left under vacuum at 95 °C for 1.5 h. After thoroughly drying, the flask was filled with nitrogen, and 200 mL of anhydrous DMF was added via a cannula while stirring. The flask was then equipped with a thermometer and heated to 95 °C while stirring the solution. Once the dextran was completely dissolved, the solution was cooled to 0 °C and anhydrous pyridine (2.0 mL, 25.8 mmol) was added. Subsequently, freshly sublimed PNC (2.5 g, 12.4 mmol) was added in small portions, keeping the temperature below 2 °C. After 1 h, the reaction mixture was poured into 1.0 L of ice-cold ethanol. The precipitate was filtered off (Por 4) and washed with copious amounts of cold ethanol and diethyl ether. After drying under vacuum, the product was obtained as a white powder (5.80 g, 89% yield, DS 26). ^1^H NMR (400 MHz, DMSO-*d*_6_): δ­(ppm) = 3.0–4.0 (saccharide ring protons, m, 6H); 4.2–5.8 (anomeric and hydroxyl protons, m, 4H); 7.58 (Ar o-CH, d, 2H); 8.34 (Ar m-CH, d, 2H).

Next, Dex-PNC with DS 26 (5.0 g, 6.33 mmol of PNC) was weighed into a 250 mL three-necked round-bottom flask equipped with a stirrer bar. The flask was filled with nitrogen, and 100 mL of anhydrous DMF was added via a cannula while stirring. Once the Dex-PNC was completely dissolved, *n*-Boc-1, 4-diaminobutane (0.483 g, 2.57 mmol) was added under a nitrogen flow and the reaction was allowed to proceed for 1 h. After 1 h, the reaction mixture was poured into 1.0 L of ice-cold ethanol. The precipitate was filtered off (Por 4) and washed with copious amounts of cold ethanol and diethyl ether. After drying under vacuum, the product (Dex-bNHBoc) was obtained as a white powder and dissolved in 50 mL of Milli-Q water. Trifluoroacetic acid (TFA) (5 mL) was added, and the mixture was stirred overnight. The reaction mixture was neutralized by 1.0 M NaOH solution and dialyzed against Milli-Q water for 4 days (MWCO 3500 Da), followed by freeze-drying yielding the product (Dex-bNH_2_) as a white foam (3.61 g, 88% yield). ^1^H NMR (400 MHz, DMSO-*d*_6_): δ­(ppm) = 1.4–1.6 (−CH_2_–(CH_2_)_2_–CH_2_NH– protons, m, 4H); 3.0–4.0 (saccharide ring protons, m, 6H); 4.2–5.4 (anomeric and hydroxyl protons, m, 4H).

Dex-Mal: Dex-bNH_2_ (2.0 g, 1.00 mmol –NH_2_) was weighed into a 100 mL three-necked round-bottom flask equipped with a stirrer bar. The flask was filled with nitrogen, and 50 mL of anhydrous DMF was added via a cannula while stirring. Once the Dex-bNH_2_ was completely dissolved, Et_3_N (0.289 g, 2.85 mmol) and AMAS (0.481 g, 1.90 mmol) were added. The mixture was stirred for 24 h at room temperature under a nitrogen atmosphere. After 24 h, the reaction mixture was poured into 500 mL of ice-cold ethanol. The precipitate was filtered off (Por 4) and washed with copious amounts of cold ethanol and diethyl ether. After drying under vacuum, the crude product was obtained as a white powder. The crude product was dissolved in water and dialyzed against Milli-Q water for 3 days (MWCO = 3500 Da). Followed by filter sterilization and freeze-drying, the product was yielded as a white foam (1.96 g, 92% yield). ^1^H NMR (400 MHz, DMSO-*d*_6_): δ 3.0–4.0 (saccharide ring protons, m, 6H); 4.2–5.4 (anomeric and hydroxyl protons, m, 4H); 6.67 (Ar m-CH, d, 2H); 6.99 (Ar o-CH, d, 2H); 7.08 (maleimide protons, d, 2H).

The calculation of the DS of –PNC, –bNH_2_, and –Mal is based on the integrals of δ 4.2–5.8 ppm (corresponding to the 4 anomeric and hydroxyl protons from dextran), compared with the integral of the aromatic 4 protons of *p*-nitrophenyl (7.40–7.65 ppm and 8.20–8.40 ppm), the 4 protons of –CH_2_–(CH_2_)_2_–CH_2_NH_2_ (1.40–1.60 ppm), or 2 protons of maleimide (7.04–7.14 ppm), respectively. The DS of dextran is given as the percentage of saccharide units modified in dextran.

### 2.4 Selection and Production of the VHHs

The monovalent VHH directed against HIV protein gp120 (anti-HIV-VHH1 or Q1c) and the bivalent VHH molecules directed against either gp120 and gp41 (J3–2E7 or Q10c) or BMP4 (16C4–16C4, C4C4, or Q35bc) were described previously and were provided by QVQ.^32–34^ The VHH binding DKK-1 (anti-DKK1-VHH) was also described previously.^35^ The VHH binding to TNF-alpha (anti-TNFα-VHH) and IL1R (anti-IL1R-VHH) was provided by Verlin. All of the VHHs were genetically fused with a C-terminal C-Direct tag containing an unpaired cysteine during cloning into the pYQ11 plasmid (previously called pYQVQ11).

At Orthros Medical BV, the plasmid DNA was transformed into Saccharomyces cerevisiae strain VWK18gal1 that was used as a host for VHH expression. The production of VHH was done as shake flask cultivation. At first, the inoculum was grown in a 250 mL baffled shake flask containing 50 mL of Difco Yeast Nitrogen Base (YNB) at 30 °C, 200 rpm for 24 h, and was subsequently transferred to 2 L baffled shake flasks with 900 mL of YPD medium (2% yeast extract, 1% peptone, 2% glucose, 1% galactose) and was grown at 30 °C, 200 rpm for 72 h.^36,37^

VHHs were purified from the yeast cell-free supernatant using an Äkta Pure (Cytiva) and Capture-Select affinity chromatography column (C-tag XL, Thermo Fisher Scientific) according to the manufacturer’s protocols. Purified VHH was filter sterilized (0.2 μm) and stored in PBS.^38^ The list of the VHHs and their characteristics is shown in Table 1.

**Table 1:** List of VHHs Used for Bioconjugation

VHH	format	antigen	MW (Da)
anti-HIV-VHH1	monospecific	HIVgp120	15,155
anti-HIV-VHH2	bivalent, monospecific	HIVgp120 and gp41	28,968
anti-DKK1-VHH	monospecific	DKK1	15,434
anti-BMP-VHH	bivalent, bispecific	BMP2 and BMP4	29,707
anti-IL1R-VHH	monospecific	IL-1R	14,863
anti-TNFα-VHH	monospecific	TNFα	15,567

### 2.5 Preparation of VHH-HA and VHH-Dex Conjugates

Before conjugation of VHHs to the polymers (HA-Mal and Dex-Mal), the VHHs were reduced in PBS, to split the VHH dimers created due to the spontaneous formation of disulfide bridges between the unpaired Cys residues introduced in the C-terminus of the VHHs. Reduction was achieved by the addition of a 10-fold molar excess of TCEP and incubation at 37 °C for 1 h, followed by the removal of TCEP through ultrafiltration units with a 3 kDa MWCO (Amicon Ultra, Millipore). A typical conjugation reaction was carried out as follows: The reduced VHHs were added to HA-Mal or Dex-Mal solution in PBS (pH 7.4) in a 1:10 molar ratio (VHH:polymer) to produce VHH-HA or VHH-Dex conjugates. The reaction mixtures were gently shaken at 4 °C overnight. The unreacted maleimide moieties were blocked by the addition of 1.5-fold molar excess of Cys. Finally, excess Cys was removed through ultrafiltration units with a 3 kDa MWCO at 4 °C with PBS buffer (pH 7.4).

### 2.6 Surface Plasmon Resonance Imaging (SPRi)

For immobilization purposes, biotinylated TNFα antibody (BioLegend, San Diego, USA) was diluted to concentrations ranging from 0.3125 to 5.0 μg/mL in sodium acetate immobilization buffer with a pH of 4.5, prepared as described previously.^39^ The immobilization on G-Strep sensors (SSens, Enschede, The Netherlands) was carried out using a continuous flow spotter (Wasatch Microfluidics, Salt Lake City, UT, US), where 48 spots were printed in 30 min. To reduce nonspecific interactions, the sensor was deactivated with Strep Blocking solution (SSens, Enschede, The Netherlands) during a 30 min association run. After immobilization and deactivation of the sensor, the analytes were diluted in a system buffer containing 0.075% Tween-80 in PBS. Regeneration was carried out using 200 mM phosphoric acid with a pH of 2.5. The IBIS MX96 (IBIS Technologies, Enschede, The Netherlands) was used for SPRi measurements. The back and forth flow was set to 10 μL/min in a flow cell containing 12 μL of sample. SprintX software was used for data collection and referencing. Data was exported and analyzed with Scrubber 2.0 software (BioLogic Software, Australia) and customized MATLAB scripts.

### 2.7 SPRi Kinetic Titration Assay

The affinity of the VHH and VHH-polymer constructs to recombinant TNFα was determined by the kinetic titration method with Rmax100 proposed by Schasfoort et al.^40^ Biotinylated TNFα antibody was immobilized on a G-Strep sensor in quadruple concentration ranges as described above, followed by a blocking step. For the SPRi measurements, first, an analyte injection with blanks (running buffer only) was injected that provided background for interaction signals. The next analyte injection consisted of a TNFα injection for 30 min association followed by a blank and a range of increasing concentrations (8.125–130 nM) of each VHH in running buffer. Each new VHH injection was proceeded by an injection with fresh TNFα. The measurements were initiated with a 1-min baseline stabilization period. This was followed by a 15-min association phase for both the blank and VHH samples, after which a 12-min dissociation phase was conducted. After each analyte injection, the sensor was regenerated using a double regeneration pulse for 30 s.

### 2.8 SDS-PAGE and Western Blot Analysis

For VHH-polymer conjugate (VHH-HA and VHH-Dex) analysis, sodium dodecyl sulfate polyacrylamide gel electrophoresis (SDS-PAGE) was carried out in a Mini-PROTEAN Tetra Cell system (Bio-Rad). This was connected to a PowerPac Basic (Bio-Rad) programmable power supply. VHH-polymer conjugates were treated with 4x Laemmli sample buffer (Bio-Rad), adding 10 wt % 2-mercaptoethanol to the buffer prior to mixing with the sample. Samples were then heated to 95 °C for 5 min to denature the protein sample and ensure reduction of any disulfide bonds. Samples were loaded onto commercially available 10-well 4–15% Mini-PROTEAN TGX Precast Protein Gels (Bio-Rad). Gels ran for 45 min at 160 V in running buffer (25 mM Tris, 192 mM glycine, 0.1% w/v SDS, pH 8.3). Protein bands were subsequently visualized by incubating the gels in a staining solution (0.1% Coomassie Brilliant Blue R 250, 40% MeOH, 10% acetic acid in water) with gentle agitation for 1 h. This was followed by a destaining protocol in which the gels were gently agitated in a destaining solution (40% MeOH, 10% acetic acid in water) for several hours, with the solution refreshing every hour until the background of the gel became fully destained. Finally, the gels were imaged by a FluorChem M system (ProteinSimple). In addition, free VHH and polymer-conjugates were identified using SDS-PAGE followed by Western Blot using rabbit anti-llama IgG antibody (1:1000 dilution, K1612, QVQ, Utrecht, The Netherlands) and secondary antibody coupled to horseradish peroxidase (1:2000 dilution, PO448, Dako, CA, USA). Immunoreactive bands were developed by using the SuperSignal Western Blot Enhancer kit (Thermo Fisher) and visualized with the FluorChem M system. The protein bands in the gels or membranes were quantified by AlphaView software (ProteinSimple).

## 3 Results and Discussion

### 3.1 Synthesis of Dex-Mal and HA-Mal

Two different polysaccharides, i.e., dextran (Dex) and hyaluronic acid (HA), were utilized to create VHH-polymer conjugates. To conjugate a free Cys-containing VHH to the polymers, maleimide groups grafted onto the polymer backbone were chosen as the thiol-reactive entities. This enabled directed coupling of the unpaired free Cys-containing VHH to the polymer backbone via stable thioether bonds. Maleimide is one of the most widely used Michael acceptors for producing protein–polymer conjugates for therapeutics at high efficiency.^20^ In this work, we successfully developed an approach for the synthesis of dextran-maleimide (Dex-Mal), which was achieved through a four-step process. Initially, the hydroxyl groups of dextran were activated using PNC to form carbonate intermediates, which were then reacted with *n*-Boc-1,4-diaminobutane. Following the removal of the Boc protecting group, the primary amine group of Dex-bNH_2_ served as a reactive site for conjugation with the NHS ester of a maleimide derivative, *n*-(α-maleimidoacetoxy) succinimide ester (AMAS), resulting in the formation of Dex-Mal, as illustrated in Figure 1A. The previously reported modification method for HA was utilized to synthesize hyaluronic acid-maleimide (HA-Mal),^29^ which involved modifying the carboxylic group on the glucuronic acid moiety of HA through a DMTMM-activated amidation reaction (Figure 1B).

The successful synthesis of dextran-maleimide (Dex-Mal) and hyaluronic acid-maleimide (HA-Mal) was confirmed by using ^1^H NMR (Figure 1C,D). The ^1^H NMR spectra indicated that the number of modified maleimide moieties per 100 monosaccharides in dextran and 100 disaccharides in hyaluronic acid was 6 and 12, respectively.

### 3.2 Conjugation of Diverse Cys-Tagged VHHs to Dex-Mal and HA-Mal

Generally, naturally occurring Cys residues in proteins are not accessible for modification since they are involved in the formation of disulfide bridges and their modification will often result in loss in protein structure and thereby decrease the bioactivity of the protein.^41,42^ Therefore, key to the present work is the introduction of a solvent-accessible unpaired Cys at the C-terminus of the protein through an additional genetic engineering step. This free Cys can easily react with maleimides on polymers. To investigate the conjugation efficiency of the different VHHs to both Dex-Mal and HA-Mal, we selected six different VHHs with an unpaired Cys at the C-terminus for conjugation (Table 1).

Conjugation occurred by incubating the VHHs with 10 equiv of HA-Mal or Dex-Mal in 100 mM PBS at 4 °C overnight (Figure 2A). Before conjugation of the VHHs to polymers, it is worth noting that an important requirement is the reduction of the disulfide bonds due to oxidation of free Cys residues. This was achieved by the use of tris­(carboxyethyl)­phosphine (TCEP), which is capable of rapidly reducing disulfide bonds at a neutral pH and is nonreactive with thiol-reactive compounds like maleimides.^43^ After conjugation, the coupling efficiency of VHH to HA-Mal or Dex-Mal was determined with SDS-PAGE or Western Blot. Western Blot has more specificity than SDS-PAGE, which allows the detection of both the conjugated VHHs and unconjugated VHHs using an anti-VHH antibody for visualization. By Western Blot analysis, VHH-HA conjugates appeared as a smear with a larger effective molecular weight than unconjugated VHH (Figure 2B). To simplify the procedure of analysis, Coomassie-stained SDS-PAGE was used to detect the VHH-Dex conjugates (Figure 2C). Subsequently, the conjugation efficiency of each VHH was expressed as a relative intensity of the band at the molecular weight of the free VHH compared to the same VHH without addition of polymer-maleimide. It was observed that the intensity of free VHH, which was not coupled to the polymer, decreases when polymer-maleimide conjugates were added to the VHH, indicating successful conjugation of VHH to polymers. Figure 2D,E shows an overview of the conjugation efficiencies of the six different VHHs to HA-Mal and Dex-Mal, respectively. Large differences in conjugation efficiency of VHHs to the same polymer were observed; however, there was no significant difference between the conjugation to HA-Mal and Dex-Mal. The VHHs anti-HIV-VHH1, anti-HIV-VHH2, and anti-IL1R-VHH show a relatively high conjugation efficiency above 70%. However, the conjugation efficiency of anti-TNFα-VHH to HA-Mal and Dex-Mal, at 7% and 5%, respectively (Figure 2D,E), was extremely low, indicating that especially the properties of the VHH itself such as the amino acid sequence, hydrophobic character, charge distribution, and potentially its three-dimensional folding, which collectively determine the accessibility of Cys residues, play a critical role in influencing the conjugation reaction. Although the chemical and physical properties of the two polysaccharide polymers, dextran (neutral) and hyaluronic acid (negatively charged), slightly influenced the conjugation of VHHs, it is more likely that accessibility of the thiol group of the unpaired Cys primarily affected the conjugation efficiency of VHHs to the maleimide-functionalized polymers. The low efficiency of conjugation of anti-TNFα-VHH possibly reflects limited solvent accessibility of the Cys residue. This limitation is commonly observed in Cys conjugation chemistries.^44^

**Figure 2:**
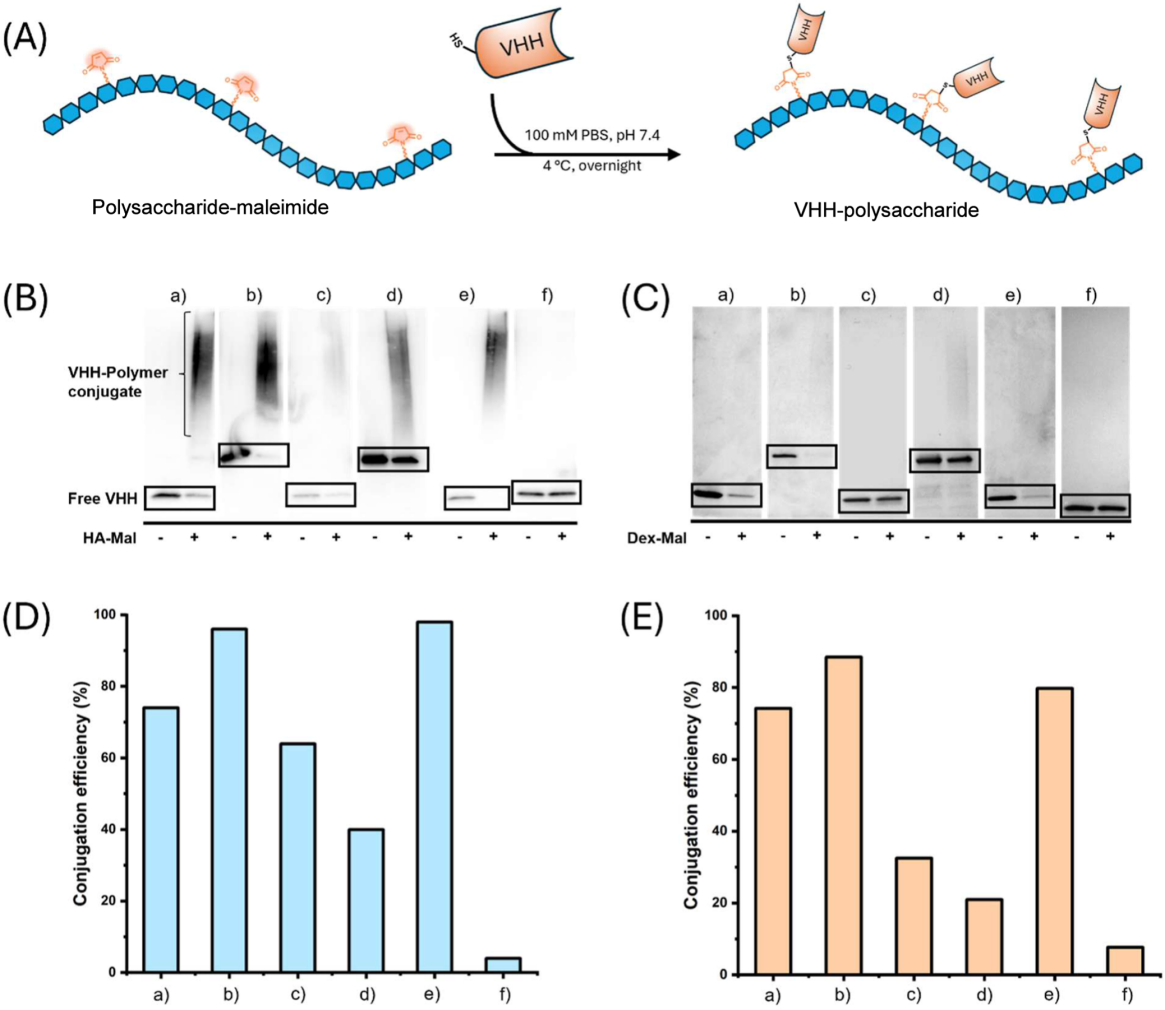
Conjugation of free Cys-containing VHH to a maleimide-functionalized polysaccharide (A). Western Blot analysis after conjugating VHHs to HA-Mal (B) and SDS-PAGE analysis after conjugating VHHs to Dex-Mal (C), showing the VHH before (−) or after (+) the conjugation reaction with the polymer. The quantified conjugation efficiency of VHHs to HA-Mal (D) and Dex-Mal (E). For all VHH conjugation reactions, conjugation efficiency was determined by the band intensity, measured via AlphaView software. Labels in (B)–(E) are corresponding to VHHs; (a) anti-HIV-VHH1-Cys, (b) anti-HIV-VHH2-Cys, (c) anti-DKK1-VHH-Cys, (d) anti-BMP-VHH-Cys, (e) anti-IL1R-VHH-Cys, and (f) anti-TNFα-VHH-Cys.

### 3.3 Effect of the Genetically Incorporated G_4_S Linker on the Binding Affinity of Anti-TNFα-VHH

In order to improve the poor coupling of anti-TNFα-VHH to the maleimide-functionalized polymers, a hydrophilic flexible linker was incorporated between the C-terminus of the VHH and the Cys tag through an additional genetic engineering step. In general, flexible linkers are rich in small or polar amino acids such as Gly and Ser to provide good solubility and flexibility.^45^ In the present work, we used a so-called G_4_S linker, whose sequence consists of four Gly and one Ser residues and can be introduced as multiple repeats, i.e., (G_4_S)_n_. The length of this G_4_S linker can be optimized to achieve an appropriate separation of the paratope of the VHH and Cys tag. It was hypothesized that this linker can enhance the accessibility of the free Cys of the VHH for the reaction with the maleimide group of the polymers. In addition, an anti-TNFα-VHH-(G_4_S)_2_-2Cys was produced by inserting a tandem repeat of the Cys tag into the C-terminus of the VHH, which provided another way to make it more accessible to couple to the polymers. The design of these three VHH constructs is shown in Figure 3A. As shown in Figure 3B, the molecular weights of these three VHHs, anti-TNFα-VHH-Cys, anti-TNFα-VHH-(G_4_S)_2_-Cys, and anti-TNFα-VHH-(G_4_S)_2_-2Cys, were characterized by SDS-PAGE analysis and were consistent with the theoretically predicted molecular weight based on their amino acid composition.

**Figure 3:**
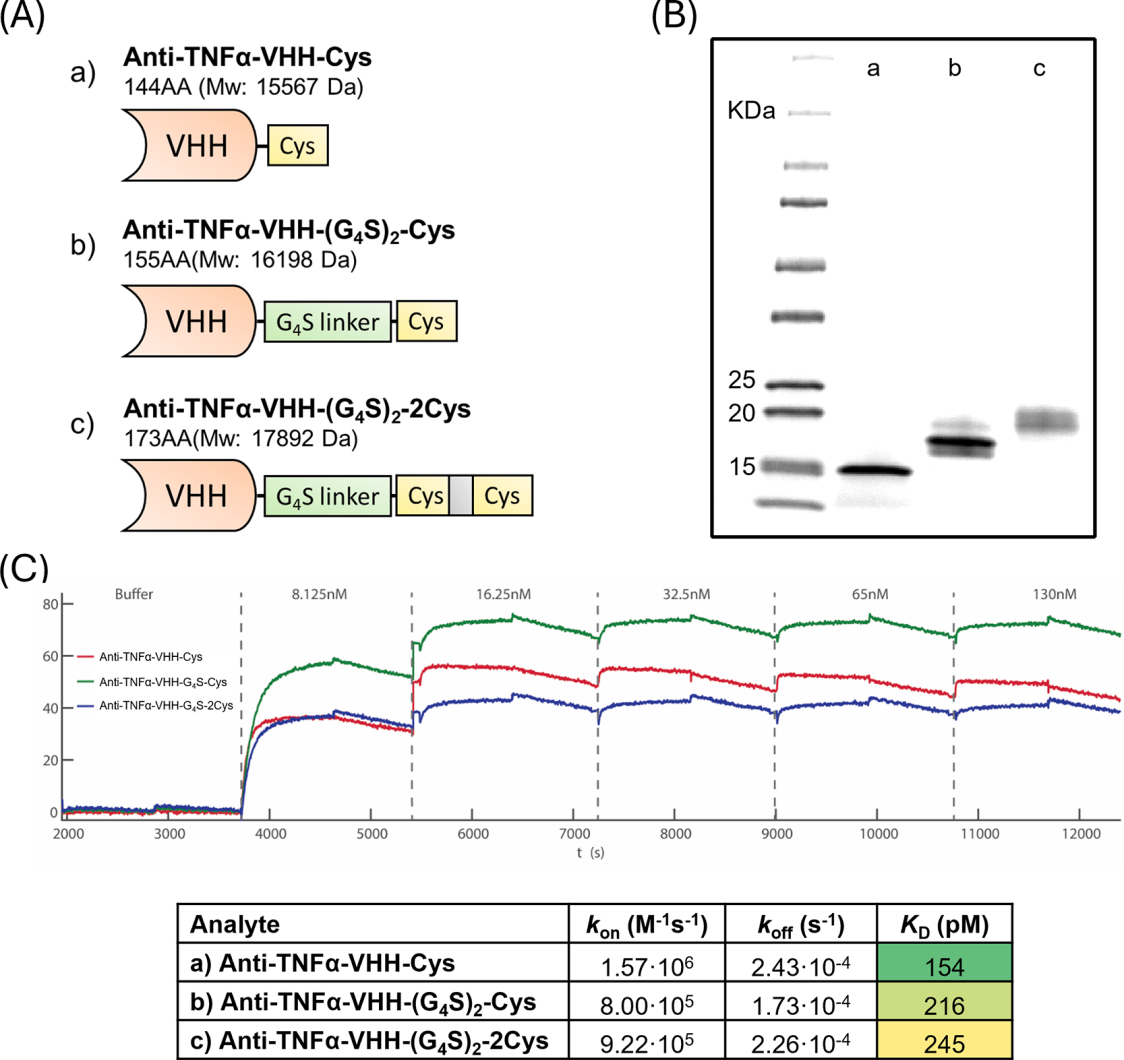
Design and characterization of the TNFα-targeting VHH constructs. Illustrations and details of the used VHH constructs (A). Cys stands for unpaired Cys. The G_4_S linker, GGGGSGGGGS, was introduced between the functional domain of the VHH and the unpaired Cys. SDS-PAGE analysis of the three different VHHs (a–c) (B). Binding kinetics of these three VHHs targeting TNFα measured by SPRi (C). The table shows the SPRi quantification.

The binding kinetics of the anti-TNFα-VHH-Cys, anti-TNFα-VHH-(G_4_S)_2_-Cys, and anti-TNFα-VHH-(G_4_S)_2_-2Cys to TNFα were characterized by using surface plasmon resonance imaging (SPRi) measurements. The equilibrium dissociation coefficient (*K*_D_), that was calculated by dividing the dissociation rate (*k*_off_) by the association rate (*k*_on_), is inversely proportional to binding affinity between antibody and antigen. The anti-TNFα-VHH-(G_4_S)_2_-Cys and anti-TNFα-VHH-(G_4_S)_2_-2Cys binding toward TNFα in SPRi showed a low *K*_D_ value of 216 and 245 pM, respectively, that corresponded with high affinity (Figure 3C). When the binding kinetics of these two VHHs were compared to the parent anti-TNFα-VHH-Cys (*K*_D_ = 154 pM), it was seen that the *k*_off_ value was similar but the *k*_on_ values for both anti-TNFα-VHH-(G_4_S)_2_-Cys and anti-TNFα-VHH-(G_4_S)_2_-2Cys were lower, indicating a slower association rate, which resulted in slightly higher overall *K*_D_ and thus a lower affinity. This indicated that the extension of the C-terminal tail had a slight impact on the binding affinity.

### 3.4 Enhancing Conjugation Efficiency via Genetic Engineering and Its Impact on Binding Affinity in Polysaccharide Conjugates

After introducing a G_4_S linker to produce anti-TNFα-VHH-(G_4_S)_2_-Cys, the conjugation of VHH to both HA-Mal and Dex-Mal was significantly enhanced, reaching 75% and 71%, respectively (Figure 4A), compared to anti-TNFα-VHH-Cys, which showed conjugation efficiencies of 7% and 5%. Moreover, introducing one more Cys tag into the C-terminus of the VHH greatly increased conjugation efficiency. An efficiency of 93% and 91% was achieved after incubating the anti-TNFα-VHH-(G_4_S)_2_-2Cys (with two free Cys) with HA-Mal and Dex-Mal, respectively. This very high coupling efficiency suggests that the length of the G_4_S linker and/or extra Cys positively influences the coupling of this VHH to maleimide-functionalized polymers. Afterward, the binding affinity of the anti-TNFα-VHH-(G_4_S)_2_-Dex and anti-TNFα-VHH-(G_4_S)_2_-HA conjugates (both based on anti-TNFα-VHH-(G_4_S)_2_-2Cys) was determined using SPRi (Figure 4B). Comparing the binding kinetics of the conjugates to the pristine anti-TNFα-VHH-(G_4_S)_2_-2Cys, it was seen that the conjugates showed a slightly higher overall *K*_D_ value and thus a lower affinity. After the VHH was coupled to the polymers, both *k*_on_ and *k*_off_ showed a slight decrease. The results indicate that the antigen is slightly less likely to bind to the VHH; however, once captured, it is also less likely to release. This suggests that multivalent interactions between VHHs and TNFα occur due to the multivalency of the VHH-polymer conjugate. In conclusion, conjugating the VHH to polymers via thiol–maleimide chemistry may slightly reduce the overall binding affinity of the VHH; nevertheless, the binding affinity of the VHH after conjugation remains sufficient for potential applications. Previously, it was demonstrated that multivalent bioconjugates of proteins could be grafted onto an HA polymer chain.^15^ Our approach has the potential to attach multiple VHHs to a single polymer chain, even with the use of low molecular weight polysaccharides. Therefore, employing high molecular weight polysaccharides could potentially further enhance the efficiency of multivalent conjugation by increasing the number of available reaction sites on the polymer chain. Moreover, multivalent conjugation within a single-chain polymer can enhance the pharmacological performance of therapeutic proteins, not only due to their larger size but also due to their increased potency.^16,46^

**Figure 4:**
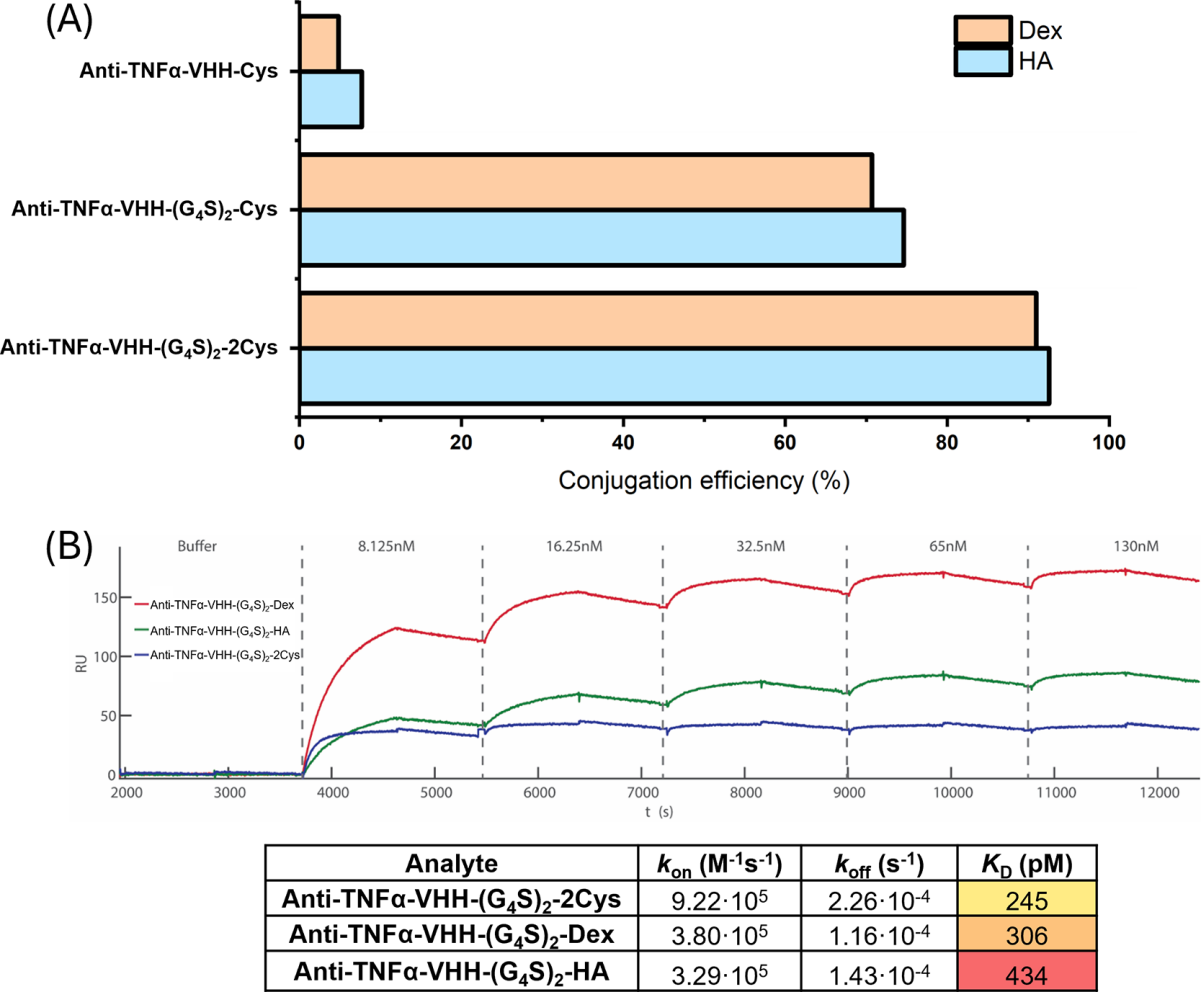
Conjugation efficiency of the TNFα targeting anti-TNFα-VHH-G_4_S-2Cys to HA-Mal and Dex-Mal was quantified by the intensity of the bands after SDS-PAGE analysis, measured via AlphaView software (A). Binding kinetics of these three VHHs targeting TNFα measured by SPRi (B). The table shows the SPRi quantification.

## 4 Conclusion

Here, we describe an effective method for creating VHH-polysaccharide conjugates based on thiol–maleimide chemistry. To achieve site-selective modification, an unpaired Cys was genetically engineered into the C-terminus of the VHH, and two maleimide-functionalized polysaccharides, Dex-Mal and HA-Mal, were synthesized for conjugation. The conjugation efficiency to these polymers depended on the identity of the VHH with some of the VHHs showing a relatively high conjugation efficiency of above 70%. However, the conjugation efficiency of one of the tested VHHs, anti-TNFα-VHH-Cys, was extremely low. Introducing a hydrophilic flexible G_4_S linker to form anti-TNFα-VHH-(G_4_S)_2_-Cys significantly improved its conjugation efficiency to the HA-Mal and Dex-Mal from 5% to 75% and 7% to 71%, respectively. Incorporation of an additional unpaired Cys at the C-terminus of anti-TNFα-VHH-(G_4_S)_2_-Cys could also significantly increase the accessibility of the Cys for the coupling reaction, resulting in a high conjugation efficiency to HA-Mal (91%) and Dex-Mal (93%). While each of these modifications resulted in a slight reduction of the binding affinity of VHH for its target protein, the affinity of the VHH-polymer conjugates was still acceptable with *K*_D_ values far below the nanomolar range. This approach for creating VHH-polysaccharide conjugates shows great potential for enhancing therapeutic performance and can serve as a complementary approach to other methods, such as hydrogel-based controlled protein delivery, to maintain effective therapeutic concentrations at the disease site.
